# Emerging Roles for Chromo Domain Proteins in Genome Organization and Cell Fate in *C. elegans*

**DOI:** 10.3389/fcell.2020.590195

**Published:** 2020-10-23

**Authors:** Abhimanyu DasGupta, Tammy L. Lee, Chengyin Li, Arneet L. Saltzman

**Affiliations:** Department of Cell & Systems Biology, University of Toronto, Toronto, ON, Canada

**Keywords:** histone methylation, chromodomain, histone reader, genome organization, epigenetic inheritance, *C. elegans*, chromatin architecture, CEC

## Abstract

In most eukaryotes, the genome is packaged with histones and other proteins to form chromatin. One of the major mechanisms for chromatin regulation is through post-translational modification of histone proteins. Recognition of these modifications by effector proteins, often dubbed histone “readers,” provides a link between the chromatin landscape and gene regulation. The diversity of histone reader proteins for each modification provides an added layer of regulatory complexity. In this review, we will focus on the roles of chromatin organization modifier (chromo) domain containing proteins in the model nematode, *Caenorhabditis elegans*. An amenability to genetic and cell biological approaches, well-studied development and a short life cycle make *C. elegans* a powerful system to investigate the diversity of chromo domain protein functions in metazoans. We will highlight recent insights into the roles of chromo domain proteins in the regulation of heterochromatin and the spatial conformation of the genome as well as their functions in cell fate, fertility, small RNA pathways and transgenerational epigenetic inheritance. The spectrum of different chromatin readers may represent a layer of regulation that integrates chromatin landscape, genome organization and gene expression.

## Introduction

Eukaryotic chromatin is highly regulated to ensure proper gene expression in different cell types and across developmental stages. The combined application of high-resolution microscopy and genome-wide sequencing approaches now provides a comprehensive view of the organization of the genome into more transcriptionally active and accessible regions of euchromatin or less active and more compact regions of heterochromatin. These chromatin states are characterized by different patterns of histone modifications and spatial separation within the nucleus [reviewed in Hildebrand and Dekker (2020)]. Understanding the interplay between the histone modification landscape and the three-dimensional conformation of the genome will provide insight into the establishment and maintenance of cell type-specific gene expression programs.

Patterns of histone post-translational modifications are associated with functionally distinct chromatin states. One key modification is methylation of lysine residues on the N-terminal tails of histone H3. The mono-, di- or tri-methylated lysine residues form a binding site for a diverse group of “reader” domains [reviewed in Patel (2016)]. These include chromo domains, which will be the focus of this review, and other members of the structurally related “Royal family,” such as tudor, PWWP and MBT (Malignant Brain Tumour) repeat domains (Maurer-Stroh et al., 2003). The two founding chromo domain families are defined by Polycomb (Pc) and Heterochromatin Protein 1 (HP1). These proteins have well-characterized and conserved roles in maintaining facultative and constitutive heterochromatin, respectively, through their recognition of methyllysine residues on histone H3 (H3K27me3 and H3K9me3) [reviewed in Eissenberg (2012)].

Chromo domain-containing proteins from diverse eukaryotes have been grouped by multiple sequence alignment into thirteen families (Tajul-Arifin et al., 2003), many of which encode chromatin modifiers and remodeling enzymes. Here we will focus on the “single chromo domain” proteins, a subset of these protein families without an accompanying catalytic domain. In *C. elegans*, these include two homologs of HP1 (HP1-Like-1, HPL-2), a homolog of the euchromatin-associated Mortality Factor-Related Gene (MRG-1), and a diverse group of *C. elegan*s chromo domain (CEC) proteins (Table 1). Additional CEC proteins have also been identified by sequence homology and await characterization (Aasland and Stewart, 1995; Agostoni et al., 1996).

**TABLE 1:** Characteristics of *C. elegans* chromodomain proteins discussed in this review.

**Protein**	**Domains**	**Histone modification interactions**		**Expression pattern**	**Similarity to human proteins**	
					**Full length: (% query coverage,% identity; OrthoList 2)**	**Chromodomain only: (% query coverage,% identity)**
HPL-1	CD, CSD	H3K9me H1K14me H3K23me	me3 (*in vitro*) me1 (*in vitro*, co-IP) me1/2/3 (*in vitro*), me2 (co-IP)	Embryo (Tg): from 50 cell stage Larva – Adult (Tg): broad, enriched in head, tail, hypodermis, and some neurons	CBX3/HP1γ (77, 36; 4) CBX5/HP1α (75, 36; 4) CBX1/HP1β (70, 34; 5)	CBX3 (88, 49) CBX5 (92, 49) CBX2 (96, 43)
HPL-2	CD, CSD	H3K9me H3K27me	me1/2/3 (*in vitro*, co-IP), me1/2 (ChIP-seq) me3 (*in vitro*), me2/3 (co-IP)	Embryo (Tg): broad, strong expression from 20-24 cell stage Adult (Tg): broad	CBX5 (47, 36; 2) CBX3 (52, 32; 2) CBX1 (45, 37; 3)	CBX5 (96, 46) CBX3 (88, 48) SUV39H1 (96, 38)
CEC-1	CD	H3K27me	me2/3 (*in vitro*)	Embryo (Tg): broad, from ∼50 cell stage Larvae-Adult: broad in soma, proximal germline	CBX2 (16, 51; 2) CBX4 (18, 47; −) CBX7 (17, 43; −)	CBX2 (98, 50) CBX4 (98, 48) CBX8 (98, 40)
CEC-3 (EAP-1)	CD	H3K9me	me1/2/3 (*in vitro*), me3 (ChIP-seq)	Embryo: broad Adult: enriched in head regions and germline	MMP8 (16, 47; 0) CDYL2 (15, 41; −)	MMP8 (98, 50) CDYL2 (90, 41)
CEC-4	CD	H3K9me	me1/2/3 (*in vitro*)	All stages (Tg): broad, enriched in muscles	CBX5 (30, 34; −)	CBX5 (92, 42)
CEC-6	CD	H3K9me H3K27me	me2/3 (*in vitro*) me2/3 (*in vitro*)	Enriched in primordial germ cells and germline	CDYL (5, 47; −) CBX7* (6, 33; −) *DB	CDYL (86, 47) CDY1 (79, 47) CBX2 (98, 35)
HERI-1 (CEC-9)	CD, Ser/Thr kinase-like	not known		Embryo: germ and soma blastomeres Larvae - Adult: primordial germ cells and germline	NRBP1 (26, 27; −) CDK2* (37, 16; −) *DB	CBX2 (39, 42) CBX8 (37, 47)
MRG-1	CD, MRG	H3K36me H3K4me	me2/3 (ChIP-seq) me3 (ChIP-seq)	Early embryo: broad Late embryo: enriched in primordial germ cells Adult: enriched in germline, neurons, intestine	MORF4L1/MRG15 (96, 26; 5) MOR4FL2/MRGX (68, 27; 2)	ARID4A (55, 52)

*Histone modification interaction and expression pattern data were collected from the publications listed below ^‡^. Expression patterns are based on GFP knock-in alleles or immunofluorescence, or transgenes where indicated (Tg). Homology searches were performed using BLASTP (Altschul et al., 1990) or DELTA-BLAST (Boratyn et al., 2012) against the human RefSeq protein database. Chromo domains were mapped using the Simple Modular Architecture Research Tool (SMART) (Letunic et al., 2015). Predicted orthologs from Ortho List 2 (Kim et al., 2018) are denoted by the number of supporting orthology-prediction programs for the indicated protein (0 denotes only supported by legacy gene set; −, not identified). CD, chromo domain; CSD, chromo shadow domain; ChIP, chromatin immunoprecipitation; *in vitro*, *in vitro* peptide binding assay; co-IP, co-immunoprecipitation; Tg, transgene; DB, DELTA-BLAST. ^‡^HPL-1, HPL-2: (Couteau et al., 2002; Schott et al., 2006; Koester-Eiserfunke and Fischle, 2011; Studencka et al., 2012a,b; Towbin et al., 2012; Garrigues et al., 2015; Vandamme et al., 2015; McMurchy et al., 2017); CEC-1: (Agostoni et al., 1996; Saltzman et al., 2018); CEC-3: (Greer et al., 2014); CEC-4: (Gonzalez-Sandoval et al., 2015); CEC-6: (Saltzman et al., 2018); HERI-1: (Perales et al., 2018); MRG-1: (Takasaki et al., 2007; Cabianca et al., 2019; Hajduskova et al., 2019).*

Numerous chromo domain-containing proteins play roles in gene regulation as part of multi-protein chromatin regulation complexes [reviewed in Eissenberg (2012)]. The two *C. elegans* HP1 homologs have both shared and distinct functions in development and fertility (Couteau et al., 2002; Schott et al., 2006; Meister et al., 2011; Studencka et al., 2012a) and physically associate with transcriptional repression complexes. HPL-1 has been found in an LSD-1/CoREST-like complex (lysine-specific demethylase-1, Corepressor for REST) (Vandamme et al., 2015). HPL-2 interacts with the zinc-finger protein LIN-13 and the H3K9me-binding MBT domain protein LIN-61, forming a complex that is part of the synthetic multi-vulva (synMuv) B group (Coustham et al., 2006; Harrison et al., 2007; Koester-Eiserfunke and Fischle, 2011; Wu et al., 2012). The synMuv B group of genes includes transcriptional repressors and chromatin-associated factors that influence cell fate decisions and were named for their role in repressing ectopic vulva formation [reviewed in Fay and Yochem (2007), Gonzalez-Aguilera et al. (2014)]. MRG-1 plays numerous roles in the germline (Takasaki et al., 2007; Dombecki et al., 2011; Gupta et al., 2015; Hajduskova et al., 2019) and interacts with several chromatin regulatory factors, including the histone methyltransferase SET-26 and the SIN (Switch Independent)-3 histone deacetylase complex (Beurton et al., 2019; Hajduskova et al., 2019). The cooperation of HPL-2 and MRG-1 with multiple regulatory pathways likely contributes to their roles in spatial genome regulation, as discussed below.

In addition to the HP1 homologs, the single chromo domain proteins recognizing heterochromatin-associated histone modifications include a diverse group of CEC proteins. The chromo domains of several CECs are highly similar to the Polycomb/Chromobox (Pc/CBX) proteins or to M-phase phosphoprotein 8 (MPHOSPH8/MPP8) (Table 1). However, outside the chromo domain, the CECs diverge from these putative homologs. In flies and mammals, Pc/CBX recognizes H3K27 methylation as part of the canonical Polycomb Repressive Complex 1 (cPRC1), which participates in the maintenance of silenced chromatin domains [reviewed in Kuroda et al. (2020)]. In human cells, MPP8 recognizes H3K9 methylation as a component of the Human Silencing Hub (HUSH) complex, which regulates heterochromatin maintenance and position effect variegation [reviewed in Timms et al. (2016)]. The interactions of CEC proteins with the methylated residues of histone tails are highly suggestive of roles in chromatin-associated complexes. However, at present, it remains to be seen if any CECs are part of PRC1- or HUSH-like complexes, or if such complexes are conserved in *C. elegans*.

**FIGURE 1:**
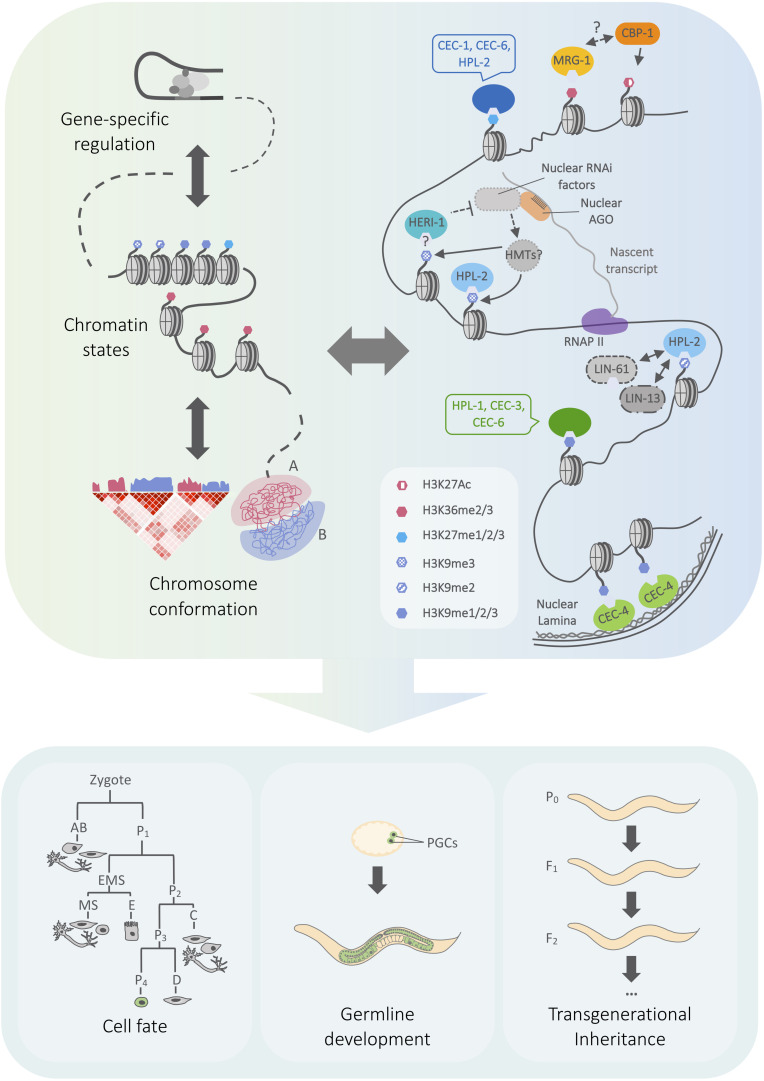
Model outlining connections between chromo domain regulation and genome architecture in *C. elegans*. **(Left)** Cartoon representing genome regulation at different scales, from transcription to chromatin state and chromosome compartmentalization. Arrows represent the mutual influence of regulatory layers. A/B compartments correspond to euchromatin and heterochromatin, which have characteristic patterns of histone modification enrichment. **(Right)** Overview of heterochromatin- and euchromatin-associated chromo domain-containing proteins, including selected physical and genetic interactions discussed in the text. Chromo domain proteins may function within a network of regulatory pathways that influence genome expression at local and global scales. **(Bottom)** Chromatin mechanisms play key roles in regulating cell fate plasticity in different developmental contexts, the maintenance of fertility, and the inheritance of small RNA-initiated silencing. See text for details on the roles of specific chromo domain proteins. Simplified embryonic lineage adapted from Sulston et al. (1983). AGO, Argonaute protein; HMT, histone methyltransferase, RNAPII, RNA polymerase II, PGC, primordial germ cell.

The recruitment and regulation of chromatin-modifying complexes are important for the establishment and maintenance of chromatin landscapes. In addition, there is a growing appreciation for the significance of three-dimensional chromosome conformation as a layer of genome organization that is interconnected with transcription and chromatin state regulation [Figure 1; reviewed in Rowley and Corces (2018)]. Examples across species point to conserved roles of heterochromatin regulators in genome topology (Klocko et al., 2016; Veluchamy et al., 2016; Falk et al., 2019), including the Pc/CBX chromodomain proteins [reviewed in Kim and Kingston (2020)]. Chromo domain proteins can therefore affect both local and global genome architecture. Recent findings reveal the importance of both of these regulatory mechanisms for *C. elegans* single chromo domain proteins.

In this review, we highlight recent studies revealing how *C. elegans* chromo domain proteins provide a connection between chromatin landscape and three-dimensional genome architecture. We also discuss the functional importance of chromo domain proteins in maintaining the balance between heterochromatin and euchromatin and the consequences for cell fate, fertility and epigenetic inheritance.

## Chromo Domain Proteins and Spatial Organization of the Genome

Across metazoans, individual chromosomes occupy discrete territories within the nucleus, and can be further separated into compartments that differ in transcriptional activity. The more active “A” compartments are gene-rich and more accessible, whereas the less active “B” compartments bear hallmarks of heterochromatin such as histone H3K9 and H3K27 methylation [reviewed in Hildebrand and Dekker (2020); Figure 1). Spatial organization, including association with the nuclear membrane-associated lamina, plays a key role in distinguishing these compartments [reviewed in Pueschel et al. (2016)]. In the *C. elegans* genome, the heterochromatic B compartments and lamina-associated domains (LADs) are enriched on the arms of the autosomes and the left end of the X chromosome [reviewed in Ahringer and Gasser (2018)]. Ostensibly, this variation in spatial localization and transcriptional activity may be influenced by the deposition and recognition of histone modifications. Indeed, recent studies have identified roles for three *C. elegans* chromo domain proteins, CEC-4, MRG-1, and HPL-2, in regulating genome architecture (see below). The application of genome-wide chromosome conformation capture (Hi-C) and chromatin immunoprecipitation assays along with high resolution microscopy approaches have together revealed cell type- and developmental stage-specific effects of chromo domain proteins on LADs, compartments and chromosome compaction.

### Lamina-Associated Domains and Compartments

The perinuclear anchoring of lamina-associated domains in *C. elegans* is facilitated by the chromo domain proteins CEC-4 and MRG-1 (Gonzalez-Sandoval et al., 2015; Cabianca et al., 2019). The role of CEC-4 was initially characterized by monitoring the localization of a heterochromatic transgene at the inner nuclear membrane using a *lacO*/lacI-GFP live imaging approach (Gonzalez-Sandoval et al., 2015). The repetitive *lacO*-containing reporter was enriched for H3K9 and H3K27 methylation and its localization was dependent on the histone H3K9 methyltransferases *met-2* and *set-25*, making it an effective readout for altered heterochromatin anchoring (Towbin et al., 2012). In embryonic cell nuclei, loss of *cec-4* disrupted lamina localization of this reporter as well as the association of the endogenous heterochromatin-enriched chromosome arms with the conserved lamin-associated protein *lem-2* (Gonzalez-Sandoval et al., 2015). Intriguingly, in the nuclei of intestinal cells in L1 larvae, MRG-1, which in contrast to CEC-4 associates with euchromatin, functioned in a partially redundant manner with CEC-4 to localize the heterochromatin reporter and chromosome arms to the nuclear lamina (Cabianca et al., 2019). Delocalization of the reporter from the lamina in *mrg-1* mutants was associated with a gain of histone acetylation, and perinuclear anchoring of the reporter could be partially rescued by depletion of the transcriptional coregulator and histone acetyltransferase CBP-1/p300 (CREB-binding protein) (Cabianca et al., 2019). These data led to a model wherein enrichment of MRG-1 and H3K36 methylation at euchromatin sequesters CBP-1 activity, consequently preventing the mistargeting of CBP-1 activity to heterochromatin, which can lead to delocalization and transcriptional derepression. Thus, pathways depending on both heterochromatin and euchromatin reader proteins have overlapping and developmental stage-specific roles in the anchoring of lamina-associated domains. Furthermore, heterochromatin anchoring is not simply driven by heterochromatin-associated factors. Their actions must be balanced by the activity of euchromatin regulators to ensure the spatial organization of heterochromatin.

Two recent studies have investigated the role of *cec-4* in genome compartmentalization using high resolution microscopy and HiC. These approaches enable complementary insights from single-chromosome and population average perspectives, respectively. In the imaging approach, chromosomes I and V were visualized by chromosome tracing, a high-throughput DNA-fluorescence *in situ* hybridization (FISH) strategy, which revealed that A/B compartments emerge upon gastrulation (Sawh et al., 2020). Prior to this stage, the most prevalent chromosome configuration in early embryos was a barbell-like shape, with more densely folded “pre-B” compartment arms and a less compact pre-A central region. When lamina attachment was disrupted by mutation of *cec-4*, chromosomes occupied less space, were more disorganized, and, in particular for the larger chromosome V, exhibited less separation between the distal pre-B arms. These results suggest that CEC-4-mediated lamina anchoring stretches the chromosomes in the early embryo (Sawh et al., 2020). The effects of anchoring may vary by chromosome, sequence, or developmental context, as *cec-4* mutation leads to decompaction of the X chromosome in differentiated cells of the adult [see below; (Snyder et al., 2016)].

To separate the contributions of lamina tethering and H3K9 methylation, the HiC study combined mutation of *cec-4* and the histone methyltransferases *met-2* and *set-25* (Bian et al., 2020), the loss of which leads to undetectable H3K9 methylation (Towbin et al., 2012; Zeller et al., 2016). Overall, CEC-4-dependent anchoring strengthened autosome compartments by enhancing the separation of the B compartment arms from the central A compartment regions and by promoting inter-chromosomal interactions among A compartments. Anchoring also promoted intra-chromosomal interactions between the distal arms (B compartments) specifically on the smaller chromosomes (I, II, III). Notably, H3K9 methylation also promoted the compaction of B compartments (intra-arm interactions), but in a *cec-4*-independent manner (Bian et al., 2020). It is plausible that HP1 homologs are effectors of this *cec-4*-independent arm compaction, as described in the context of small RNA regulation [see below; Fields and Kennedy (2019)]. Moreover, loss of H3K9me did not eliminate compartments, leaving the door open for other chromatin pathways.

### Chromatin Compaction in Dosage Compensation and Nuclear RNA Interference

In addition to lamina association, chromosome compaction is a key feature of genome architecture that is mediated by chromo domain proteins in coordination with other pathways. In *C. elegans* hermaphrodites, X chromosome compaction is one of the mechanisms through which the dosage compensation complex (DCC) facilitates downregulation of the two X chromosomes in the soma [reviewed in Albritton and Ercan (2018)]. This compaction was assayed by X chromosome-paint DNA-FISH and found to depend on the nuclear lamina-anchoring factor *cec-4* and several histone methyltransferases including the H3K9 methyltransferases *met-2* and *set-25* (Snyder et al., 2016). Surprisingly, in *cec-4* mutant animals, the heterochromatic left domain of the X chromosome remained anchored, whereas the more gene-rich euchromatic regions exhibited more pronounced decondensation and aberrant central localization in the nucleus. Therefore, at least in the context of the dosage-compensated X chromosome, CEC-4 facilitates compaction of euchromatic regions, in addition to its role in anchoring heterochromatin at the nuclear lamina (Gonzalez-Sandoval et al., 2015). Although loss of *cec-4* had limited effects on gene expression in embryos (Gonzalez-Sandoval et al., 2015), there was a subtle but significant upregulation of genes on the X chromosome in L1 larvae, a timepoint when dosage compensation is normally fully established (Snyder et al., 2016). Thus, compaction is one of several mechanisms important in dosage compensation. It will also be of interest to determine the potential relationships among *cec-4*-dependent compaction, the parallel *mrg-1*-dependent mechanism described above (Cabianca et al., 2019), and additional chromatin factors implicated in the spatial regulation of the X chromosome (Crane et al., 2015; Brejc et al., 2017; Weiser et al., 2017).

The interplay between chromo domain proteins and genome architecture is further illustrated by the role of *hpl-2* in nuclear RNA interference (RNAi)-mediated chromatin compaction. Small interfering RNAs (siRNAs) can direct cytoplasmic silencing that targets mRNA or nuclear co-/transcriptional gene silencing that targets the genomic locus (Figure 1). Nuclear RNAi is accompanied by deposition of histone H3K9me3 and H3K27me3 (Guang et al., 2010; Gu et al., 2012; Luteijn et al., 2012; Mao et al., 2015) and chromatin compaction that is dependent on chromatin remodelers and nuclear RNAi (NRDE) factors (Weiser et al., 2017; Fields and Kennedy, 2019). The HP1 homolog *hpl-2* has been implicated in the maintenance of nuclear RNAi-induced transcriptional silencing in the germline (Ashe et al., 2012; Shirayama et al., 2012) and the soma (Grishok et al., 2005; Juang et al., 2013). A role for *hpl-2* in nuclear RNAi-mediated compaction was demonstrated using a DNA-FISH approach to assess the spatial distribution of an integrated repetitive transgene that was targeted by nuclear RNAi (Fields and Kennedy, 2019). Notably, HP1-related proteins play conserved roles in heterochromatin regulation. HPL-2-mediated compaction may likewise involve nucleosome bridging through its chromo shadow domain, phase separation, or other compaction mechanisms (Erdel et al., 2020) [reviewed in Sanulli and Narlikar (2020)]. In addition, *hpl-2* might interact with other chromatin readers and pathways, such as factors involved in H3K27 methylation, which also mediate compaction in terminally differentiated hypodermal cells (Fields et al., 2019) and during embryogenesis (Yuzyuk et al., 2009). Although *hpl-2* was dispensable for X chromosome compaction in adult cells (Snyder et al., 2016), it will be of interest to investigate the role of compaction at other HPL-2-bound sites and in H3K9 methylation-mediated genome compartmentalization (see above) (Bian et al., 2020).

The mechanisms and biological significance of the spatial organization of metazoan genomes remain exciting and active areas of investigation. The studies above indicate numerous connections between chromo domain proteins, H3K9 methylation and genome topology. Beyond chromatin readers, higher-order chromosome structure has also been implicated in stress response and lifespan regulation in *C. elegans* (Anderson et al., 2019; Fields et al., 2019). The investigation of LADs in *C. elegans* has also made it a powerful and tractable model for understanding the mechanisms of human disease caused by lamin protein dysfunction (Harr et al., 2020).

## Functional Consequences of Regulation by Chromo Domain Proteins

### Chromo Domain Proteins in the Maintenance of Cell Fate

In metazoan development, coordinated regulation of transcription and chromatin architecture is important for the transition from cell fate plasticity to commitment [reviewed in Yadav et al. (2018)]. During *C. elegans* embryogenesis, the transition to a more differentiated state is accompanied by a progressive increase in chromatin compaction (Mutlu et al., 2018; Costello and Petrella, 2019). Furthermore, multiple chromatin-based mechanisms, including both repressive and activating chromatin-modification and chromatin remodeling activities, ensure proper cell-type- and developmental-stage-specific gene expression in the germline and soma (Cui et al., 2006; Petrella et al., 2011; Wu et al., 2012; Rechtsteiner et al., 2019) [reviewed in Robert et al. (2015)]. Thus, a network of chromatin-associated factors governs the maintenance of cell fate in *C. elegans.*

Cell fate maintenance can be countered by both naturally-occurring cell fate conversions (transdifferentiation) and experimentally-induced reprogramming. Ectopic expression of cell fate-determining transcription factors in *C. elegans* has revealed an important role for histone modification pathways [reviewed in Rothman and Jarriault (2019)]. Chromo domain proteins can modulate the susceptibility of embryonic and differentiated cells to induced reprogramming (see below). These findings highlight the roles of chromo domain proteins in linking chromatin organization to transcriptional regulation and cell fate.

In early development, the blastomeres of the *C. elegans* embryo are susceptible to cell fate conversion by forced expression of the transcription factor HLH-1, the homolog of the master regulator of myogenesis, MyoD [reviewed in Rothman and Jarriault (2019)]. This assay revealed that *cec-4* mutant embryos were less susceptible than wild-type to ectopic cell fate reprogramming (Gonzalez-Sandoval et al., 2015). Whereas all wild-type embryos were reprogrammed to muscle, ∼25% of *cec-4* mutant embryos hatched. However, this “escape” from induced muscle fate was incomplete, as these hatched embryos were fragile, expressed muscle markers ectopically, and did not continue to develop further. As discussed above, CEC-4 facilitates H3K9me-dependent anchoring of heterochromatin at the nuclear lamina and influences chromatin compartmentalization. These findings suggest that CEC-4-dependent spatial regulation is important for repression of non-induced developmental programs, and therefore that the cells in *cec-4* mutant embryos did not fully commit to the induced muscle fate (Gonzalez-Sandoval et al., 2015).

In contrast to CEC-4, the histone methyltransferases MES-2 (H3K27me) (Yuzyuk et al., 2009) and MET-2 (H3K9me2) (Mutlu et al., 2019) promoted the loss of cell fate plasticity, as the mutant embryos were more susceptible than wild-type to reprogramming. The contrasting mutant phenotypes of *cec-4* and *met-2* suggest that a CEC-4-independent function, such as impaired heterochromatin compaction (Mutlu et al., 2018), is relevant for the increased plasticity in *met-2* mutant embryos. However, it is difficult to directly compare the effects of *cec-4* and *met-2* mutations, as different embryonic timepoints and readouts for plasticity were examined. Since CEC-4, MET-2, and MES-2 all affect genome organization during embryogenesis, analysis of combinations of mutants in parallel will help to decipher whether they also regulate plasticity through similar pathways.

In contrast to the early embryo, differentiated cells lose plasticity and become more resistant to induced reprogramming. In mitotic germ cells and cholinergic motor neurons, this barrier can be overcome following loss of *mrg-1* or the HP1 homologs, respectively, indicating roles for these chromo domain proteins in protecting cell identity. When the gustatory neuron fate-inducing transcription factor CHE-1 is ectopically expressed from a heat shock responsive promoter, knockdown of *mrg-1* results in ∼25% of animals exhibiting “converted” germ cells, whereas control animals did not have converted germ cells. The conversion was assayed by expression of a fluorescent reporter for a neuronal CHE-1 target (the chemoreceptor GCY-5) and converted germ cells also developed axon-like projections (Hajduskova et al., 2019). In contrast to other factors which sensitize germ cells to CHE-1-mediated neuronal reprogramming, such as Polycomb Repressive Complex 2 (PRC2) components which regulate H3K27 methylation (Patel et al., 2012), the genomic binding sites of MRG-1 are enriched for marks of active chromatin, and MRG-1 appears to function independently of PRC2 in reprogramming (Hajduskova et al., 2019). Interestingly, MRG-1 physically interacts with the SET domain protein SET-26, which has *in vitro* H3K9 methyltransferase activity (Greer et al., 2014) and mutation of *set-26* increases the efficiency of MRG-1-mediated reprogramming. Thus, MRG-1 and SET-26 might work together through a histone methylation read-write crosstalk mechanism [reviewed in Zhang et al. (2015)] to protect germ cell fate and fertility.

Similar to the effects of *mrg-1* in the germline, *hpl-1*, *hpl-2* and heterochromatin pathways restrict the plasticity of post-mitotic cholinergic motor neurons (Patel and Hobert, 2017). When CHE-1 is induced at the last larval stage (L4), loss of both *hpl-1* and *hpl-2* led to a more robust increase in reprogramming than either alone, as measured by the number of neurons reprogrammed per animal by expression of a *gcy-5* reporter. Interestingly, the effects of *hpl-1* and *hpl-2* were partly H3K9 methylation-independent, as the efficiency of reprogramming was higher in *hpl-1;hpl-2* double mutants than in *met-2;set-25* mutants. Notably, loss of the cholinergic cell fate-determining transcription factor, *unc-3*, also sensitized these neurons to reprogramming. Combinatorial mutations indicated that *unc-3* acts in the same pathway as *met-2* but in parallel to *mes-2* and H3K27 methylation (Patel and Hobert, 2017). Collectively, these data highlight the interplay between heterochromatin-associated factors and transcription factors in specifying cell fate.

These experimental reprogramming studies reveal the roles of chromo domain proteins in connecting chromatin landscape with developmental plasticity. While studies discussed earlier focused on global chromatin reorganization, local effects on gene regulation likely also contribute to the roles of chromo domain proteins in cell fate maintenance. Indeed, fluorescent reporter assays revealed roles for H3K9 methylation readers in restricting the expression patterns of key transcription factors. For example, *hpl-1* and *hpl-2* prevent ectopic expression of reporters for homeodomain transcription factors important for male tail, vulval and gonad development (Coustham et al., 2006; Schott et al., 2006; Studencka et al., 2012b). In addition, loss of either *cec-3* or *hpl-2* leads to ectopic expression of the homeodomain transcription factor *unc-4* in non-vulval ventral nerve cord neurons and disrupted egg laying behavior (Zheng et al., 2013). These reporter assays do not reveal direct effects at the genomic loci of interest. However, the correspondence between the reporter assays and phenotypic readouts suggests that the reporters effectively model the chromo domain-dependent regulation of loci encoding transcription factors with key roles in cell fate.

Together, the cell fate induction experiments described above have revealed roles for both heterochromatin and euchromatin-associated factors in the regulation of cell fate plasticity in several developmental contexts and cell types. Looking beyond *C. elegans*, chromatin-based mechanisms have also been identified as key barriers to the reprogramming of mammalian cells [reviewed in Brumbaugh et al. (2019)]. Robust characterization of the epigenetic mechanisms governing cell fate therefore holds promise to influence advancements in regenerative medicine. One fruitful avenue will be to take advantage of the screening capabilities of *C. elegans* to identify modifiers of chromatin factor-mediated reprogramming. Such efforts have already identified connections between H3K27 methylation, the highly conserved Notch signaling pathway, and control of cell proliferation (Seelk et al., 2016; Coraggio et al., 2019). Another challenge will be to determine the mechanisms underlying cell type-specific reprogramming, and to connect the cell fate phenotypes to broad disruption of chromatin organization, or to misregulation of specific target genes. These approaches will provide a more complete understanding of the molecular networks governing cell fate plasticity.

### Germline Immortality and Transgenerational Epigenetic Inheritance

Given the importance of chromo domain proteins in cell fate, it is not surprising that they also play key roles in germ cells and fertility. Chromatin regulation affects several of the inter-related mechanisms that jointly contribute to the maintenance of the germ lineage, including the preservation of germ cell fate, repression of transposable/repetitive elements, and genome stability [reviewed in Smelick and Ahmed (2005), Kelly (2014)]. In addition, the interplay between chromatin architecture and small RNA pathways exerts a significant role in the characteristic “immortality” of the germline, or its capacity to indefinitely give rise to gametes transgenerationally. The short generation time and genetic tractability of *C. elegans* have made it a powerful model to study the mechanisms of germline immortality as well as the related phenomenon of transgenerational epigenetic inheritance (TEI), or the retention of epigenetic information across multiple generations. Here we highlight recent studies that connect chromo domain proteins to the network of mechanisms linking fertility, germline immortality and TEI.

A key model for understanding TEI in *C. elegans* is the inheritance of RNA interference (RNAi). Gene silencing initiated by RNAi can be inherited for several or many generations in the absence of the initial RNA trigger, with the duration depending on the specific pathway of silencing initiation and the nature of the genetic target [reviewed in Minkina and Hunter (2018)]. The maintenance of this silencing depends on nuclear RNAi which involves small RNA-mediated recruitment of nuclear Argonaute proteins to target loci to effect transcriptional silencing and deposition of repressive histone methylation (Figure 1) [reviewed in Weiser and Kim (2019)]. Further emphasizing the importance of TEI pathways in fertility, loss of factors essential for RNAi inheritance, including the nuclear Argonaute HRDE-1, also have a “mortal germline” phenotype (Buckley et al., 2012; Spracklin et al., 2017).

In a genetic screen for factors that prolong the transgenerational retention of RNAi inheritance, a recent study characterized the chromo domain protein HERI-1 (heritable enhancer of RNAi; formerly known as CEC-9) (Perales et al., 2018). Interestingly, ChIP assays revealed recruitment of HERI-1 to genes undergoing nuclear RNAi; this recruitment is dependent on HRDE-1 and SET-32 (also known as HRDE-3), a methyltransferase contributing to H3K9 methylation and nuclear RNAi inheritance. Together with evidence that HERI-1 inhibits nuclear RNAi, these data suggest that the silencing machinery itself recruits HERI-1 as an inhibitor, potentially forming a negative feedback loop to prevent runaway heritable epigenetic silencing. This “braking” activity may be crucial for sperm development, as *heri-1* mutants exhibit impaired spermatogenesis, which was suppressed by mutation of *hrde-1*. It will be of great interest to identify the endogenous targets of HERI-1. Additional intriguing mechanistic questions include whether its chromo domain directly interacts with methylated histones, and the potential function of its serine-threonine kinase-like domain as an allosteric regulator or scaffold (Perales et al., 2018). While much attention has been directed to the factors required for RNAi inheritance, HERI-1 joins a handful of genes or environmental perturbations identified so far that restrict TEI (Houri-Ze’evi et al., 2016; Lev et al., 2017).

The maintenance of germline immortality requires the concerted activity of multiple histone methyltransferases and demethylases [reviewed in Kelly (2014)], and genetic interaction approaches have uncovered contributions of chromo domain proteins CEC-3 and CEC-6 to this network (Greer et al., 2014; Saltzman et al., 2018). Loss of the H3K9 methylation reader *cec-3* has distinct effects in different backgrounds with compromised fertility. Strains with a mutation of the H3K4me2 demethylase *spr-5* have a mortal germline phenotype (Katz et al., 2009) which can be suppressed by a *cec-3* deletion (Greer et al., 2014). In stark contrast, *cec-6* mutants have a comparatively mild fertility defect that is sharply exacerbated in combination with loss of *cec-3* (Saltzman et al., 2018). One attractive model to account for these progressive fertility defects posits that disruption of these chromatin factors permits the aberrant spreading of transcriptionally-active euchromatin into transcriptionally-silenced heterochromatin, or vice versa, consequently disrupting germline-specific programming. Indeed, *spr-5* mutants exhibit a global increase in H3K4 methylation and a decrease in H3K9 methylation, which are associated with a progressive loss of CEC-3 association with the heterochromatin-enriched chromosome arms. In a similar manner, the ATPase MORC-1, which is required for germline immortality and RNAi inheritance (Spracklin et al., 2017; Weiser et al., 2017), also prevents the spread of H3K36 methylation into heterochromatin, and this effect can be counteracted by loss of the H3K36 methyltransferase *met-1* (Weiser et al., 2017). In the case of the *cec-3;cec-6* double mutants, the loss of both of these H3K9me and H3K27me readers may eliminate the capacity for compensatory heterochromatin recognition. However, this hypothesis remains to be tested. Further identification of both physical and genetic interactors of chromo domain proteins will help to reveal the molecular details of these models.

In addition to transgenerational effects, chromo domain proteins directly influence the development of the germline and gametes through several mechanisms. Loss of *hpl-2* results in temperature-sensitive sterility, abnormal oocyte accumulation (Couteau et al., 2002) and upregulation of repetitive elements such as transposons (McMurchy et al., 2017). The germlines of *hpl-2* mutants also exhibit hypersensitivity to DNA damage and increased apoptosis (McMurchy et al., 2017). Brood sizes of *hpl-2* mutants are further reduced by loss of additional heterochromatin factors that exhibit significant overlap in their genomic binding patterns with HPL-2 (including the synMuv factors LIN-61, LIN-13, MET-2, and LET-418), particularly at H3K9me2-marked heterochromatin and repetitive elements (McMurchy et al., 2017). These findings suggest that HPL-2 is part of a network of heterochromatin-associated proteins, including the H3K9me2 methyltransferase MET-2, that safeguard genome integrity in the germline (Zeller et al., 2016; McMurchy et al., 2017). The fertility-associated role of chromo domains in genome stability also extends to the euchromatin-associated MRG-1, which is implicated in DNA repair during meiosis and in the primordial germ cells (Xu et al., 2012; Miwa et al., 2019). Together, these studies emphasize the importance of chromo domain proteins in repetitive element and transposon repression and the response to genotoxic stress and DNA damage, in addition to their roles in gene expression regulation.

The mechanisms through which chromo domain proteins maintain germline immortality continue to be investigated. Several mortal germline phenotypes described here are reversible or temperature-sensitive (Spracklin et al., 2017; Saltzman et al., 2018), implicating epigenetic mechanisms such as the remodeling of chromatin states between generations or small RNA-based inheritance. However, given the importance of heterochromatin maintenance in genome stability [described above and reviewed in Janssen et al. (2018)], the contribution of genetic changes to this loss of fertility remains an open question.

Overall, these studies highlight the roles of multiple *C. elegans* chromo domain proteins at the intersection of chromatin architecture, small RNA pathways, and transgenerational epigenetic inheritance. *C. elegans* has also become an important model system for the transgenerational influences of environmental factors [reviewed in Perez and Lehner (2019)]. Given the associations among epigenetic mechanisms, environmental effects, aging and cancer [reviewed in Cavalli and Heard (2019)], studies in accessible model systems such as *C. elegans* are a crucial step toward a mechanistic understanding of epigenetic regulation in health and disease.

## Perspective and Future Directions

The mechanistic interplay between chromatin domain proteins, regulated expression of individual genes and three-dimensional chromatin architecture remains a pressing open question. Chromo domain proteins are particularly suited to facilitate this interplay, as they recognize histone modifications that define chromatin domains. In this review, we have highlighted evidence from *C. elegans* for heterochromatin- and euchromatin-associated chromo domain proteins directly and indirectly regulating lamina association, compaction, and maintenance of A/B compartments and chromatin domains. An emerging theme is that these chromo domain proteins operate within a network of chromatin-associated factors, transcriptional regulators, and small RNA pathways and that they may simultaneously impact multiple layers of gene regulation (Figure 1). Characterizing this diversity of function will be crucial for understanding the integration of chromatin architecture and gene expression in developmental regulation.

Investigating the mechanisms that establish and maintain the cell- and developmental stage-specific genome association patterns of *C. elegans* chromo domain proteins will shed light on the broad question of how the context-specific activities of chromatin regulation complexes are determined. Crucially, the chromatin association of proteins with “reader” domains is likely to be regulated by a combination of factors in addition to the interaction with modified histone tails. An intriguing example is provided by HPL-2, whose genomic enrichment at heterochromatic chromosome arms is reduced but not eliminated in animals lacking H3K9 methylation (Garrigues et al., 2015). Interactions with its binding partners, including the synMuv factors, may play a role in the targeting of HPL-2 to heterochromatin (Kudron et al., 2013; McMurchy et al., 2017; Saldi et al., 2018). Mechanisms regulating the association of chromo domain proteins with the genome may encompass interactions with the transcription machinery, transcription factors and RNA binding proteins, as well as direct interactions with nucleic acids [reviewed in Hiragami-Hamada and Fischle (2014), Weaver et al. (2018)]. To probe this regulatory complexity in a multicellular organism will require techniques capable of interrogating chromatin association in a tissue-specific manner [e.g., (Steiner et al., 2012; Aughey et al., 2019; Han et al., 2019)], as well as genetic analysis to identify modifiers of these patterns. A detailed mechanistic understanding will further entail a more complete picture of physical interactions of specific chromo domain proteins with other gene regulatory factors.

Another fundamental question concerns the mechanisms that maintain boundaries between active and inactive chromatin domains [reviewed in Carelli et al. (2017)]. Antagonism between chromatin modifiers with opposing functionalities (H3K27 and H3K36 methylation) plays an established role in *C. elegans* germ cell fate (Gaydos et al., 2012). In addition, disrupting multiple chromatin modification and remodeling pathways can result in cumulative, multi-generational effects on chromatin states, fertility and lifespan [reviewed in Perez and Lehner (2019)]. Emerging evidence for chromo domain proteins such as CEC-3, CEC-6, and HERI-1 as modifiers of transgenerational phenotypes suggests that these proteins might play a role in maintaining heterochromatin boundaries, perhaps by recruitment of competing histone modification machinery or transcriptional regulators, or effects on histone turnover. Such regulation of chromatin states may also impact three-dimensional genome organization. Applying chromatin conformation capture-based assays [e.g., HiC, HiChIP, reviewed in Grob and Cavalli (2018)] in additional chromo domain mutant backgrounds will help to address these questions and build on recent findings on the roles of CEC-4 and MRG-1 in chromosome topology. Such studies may also provide new insight into the forces shaping genome architecture in *C. elegans*, which lacks the key insulator and architectural protein CTCF (Heger et al., 2012).

Finally, distinguishing the functional relevance of large-scale chromatin architecture and discrete or locus-specific regulation in the phenotypes described here is an important but challenging goal. Addressing these mechanisms will likely involve the experimental manipulation of the genome and epigenome [reviewed in Holtzman and Gersbach (2018)] in combination with innovative genome-wide and imaging approaches. Indeed, coordination across local and global scales may be a key feature of the regulatory networks involved in the establishment, maintenance and resetting of cell fate in metazoan organisms. Studies in *C. elegans* and other model systems will undoubtedly continue to provide fundamental insight into these aspects of genome organization.

## Author Contributions

All authors wrote the manuscript and contributed to the article and approved the submitted version. TL and CL prepared the figure and table.

## Conflict of Interest

The authors declare that the research was conducted in the absence of any commercial or financial relationships that could be construed as a potential conflict of interest.
